# Cognition and language of 2-year-old very preterm children measured using brief validated parental report instruments: a cross-sectional study

**DOI:** 10.1007/s00431-026-07163-3

**Published:** 2026-06-23

**Authors:** Anna Markkula, Riikka Pyhälä, Petriina Munck, Jadwiga Buchwald, Marjo Metsäranta, Suvi Stolt

**Affiliations:** 1 Department of Speech-Language Pathology, Faculty of Medicine, University of Helsinki, PL 21, Haartmaninkatu 3, 00014 Helsinki, Finland; 2 Obstetrics and Gynaecology, Helsinki University Hospital and University of Helsinki, Helsinki, Finland; 3 Department of Psychology, Faculty of Medicine, University of Helsinki, Helsinki, Finland; 4 Biostatistics Unit, Department of Public Health, University of Helsinki, Helsinki, Finland; 5 Department of Pediatrics, Pediatric Research Center, New Children’s Hospital, University of Helsinki and Helsinki University Hospital, Helsinki, Finland

**Keywords:** Cognition, Language, Very preterm children, Sex difference, Brief validated parental report instruments

## Abstract

The purpose of this study is to assess the cognitive and language skills of very preterm (VP) children using brief parental report instruments at age 2 years (corrected age), potential sex differences in children’s skills, and differences between four profile groups of typical/weak cognition/language. Cognitive and language skills of 110 VP children without major neurological impairment from Finnish-speaking families were assessed using brief validated parental report instruments (cognitive scale of PARCA-R, FinCDI-SF, sentence complexity subscale of PARCA-R, CSBS-DP-ITC). Based on norms, the prevalence of weak skills (< 10th percentile) varied across cognitive and language domains between 16 and 34%. Typical development was found in 47% of the participants, while 12% showed weak skills in both domains. Weak cognition with typical language was found in 19% and typical cognition with weak language in 22% of the children. While boys and girls did not differ in raw scores, girls more frequently had weak cognition and typical language in norm-based profile groups. Furthermore, the amount of perinatal risk factors and medical complications differed in profile groups of typical/weak cognition/language.

*Conclusion*: Brief parental report instruments are useful in identifying weak cognitive and language development of VP children at age 2 years. The missing sex difference indicates that sex-specific developmental trajectories may not be applicable in VP children’s population at this age. Weak skills can occur in cognition or language, highlighting the importance of separate assessment. More research is needed on the influence of background characteristics in profile groups based on cognition and language.

**Table:** 

**What is Known:** • *Very preterm children have a high risk for cognitive and linguistic delays.* • *Brief parental report instruments are efficient and valid in detecting delayed development, but more knowledge is needed on their utility in preterm populations.*	
**What is New:** • *Brief parental report instruments are useful in detecting delayed development among 2-year-old very preterm children.* • *No sex differences could be detected in cognitive or language development. Approximately 40% of very preterm children had weak skills in only either cognition or language, highlighting the importance of assessing them separately.*

**Supplementary Information:**

The online version contains supplementary material available at 10.1007/s00431-026-07163-3.

## Introduction

Children born very preterm (VP; < 32 gestational weeks, birth weight ≤ 1500 g, or both) have a greater risk for delays in cognitive [1] and linguistic [2] development than full-term children. These risks are often explained by lower gestational age [3, 4], being small for gestational age (SGA) [5–7], and medical complications (e.g., bronchopulmonary dysplasia [1], severe intraventricular hemorrhage [8]). In addition, male sex has been reported to increase the risk for cognitive and linguistic delays among preterm children in some [8–10] but not all studies [11–13]. Further, a sex difference favoring girls in cognitive development [14–16] and particularly in language development [17] has commonly been reported in full-term children, while this has not always been observed in VP children [11, 12, 18]; thus, more research on potential sex difference in early cognition and language of VP children is needed. Further, although language problems in VP children are often suggested to reflect general cognitive deficits [19, 20], cognitive scores have weak sensitivity in identifying language difficulties [21], and it is not clear how these two developmental domains coappear in VP children during the early years of life.

Parental report instruments have been increasingly used to assess children’s early cognitive and language development [22, 23]. They are time- and cost-efficient [24, 25], have good predictive value [26–29], and provide information about skills that are not detectable in formal and time-restricted observations [30, 31]. Importantly, observations and face-to-face tests do not always provide valid information due to shyness or tiredness of the children [32]. The long-form versions of the well-known and valid parental report instrument, communicative development inventories (CDI) [33, 34], have been used to assess the language skills of preterm children in many studies [3, 11, 35–37]. However, less information is available on the usability of briefer parental report instruments such as the Infant–Toddler Checklist of Symbolic Behavior Scales Developmental Profile–Infant Toddler Checklist (CSBS-DP-ITC) [38] or short-form versions of the CDI (CDI-SF [39]) in preterm children, although they might be useful in clinical work to screen early language abilities of preterm children as they take less time to complete. In addition, another relatively brief instrument, the Parent Report of Children’s Abilities–Revised (PARCA-R) [40] can assess cognitive development of preterm children. All these brief instruments have been validated and normed. Furthermore, the development of preterm children is often followed up to 2 years, but language skills are not always assessed specifically [29].

The general aim of this study was to examine the cognitive and language development of 2-year-old VP children using brief validated parental report instruments and to evaluate their utility in detecting delayed development. The specific research questions were the following:

1. What is the level of cognitive and language skills among Finnish VP children at 2 years of corrected age (CA) measured using brief validated parental report instruments (cognitive scale of PARCA-R, Finnish short-form of CDI [FinCDI-SF], sentence complexity subscale of PARCA-R, CSBS-DP-ITC) and are there sex differences?
2. How can profile groups based on typical/weak cognition/language be identified using brief parental report instruments and what are the proportions of participants in the identified profile groups?
3. Do different background characteristics predict membership in a certain profile group of typical/weak cognition/language?

## Methods

### Participants

Participants were part of the study *Clear Path for the Language Development of Very Preterm Children*. All VP children treated in the Helsinki University Hospital and born between April 2020 and June 2022 without major neurological impairment (cerebral palsy, severe hearing impairment, blindness, major anomaly, severe diagnosed syndrome) from Finnish-speaking families (language at home > 70% Finnish) were invited. Children of parents with known alcohol or drug abuse were excluded. Recruitment occurred between June 2022 and August 2024 when the VP children followed at Helsinki University Hospital approached 2 years of CA. Eligible families were contacted via telephone by a nurse from the Neonatal Intensive Care Unit. Electronic forms of brief parental report instruments were sent to the family via the secure web application REDCap. The final sample included 110 VP children (Supplementary Fig. 1). Background characteristics of the participants were collected from the hospital records (Table 1). No sex differences were detected (*p* > 0.05).

**Table 1:** Background characteristics for all participants (*N* = 110) and for boys (*n* = 49) and girls (*n* = 61) separately. Numbers and percentages are shown. If means and SD are presented, they are indicated separately. Descriptive statistics were calculated from the available data. In case of missing data, the number of participants with available data is presented

Characteristic	*n* (%) / *M* (SD) for full sample	Min.–max for full sample	*n* (%) / *M* (SD) for boys	*n* (%) / *M* (SD) for girls
Gestational age (weeks), *M* (SD)	29.8 (2.3)	22.6–35.0	29.8 (2.3)	29.9 (2.4)
Birth weight (grams), *M* (SD)	1297.9 (358.6)	500–2380	1296.7 (347.2)	1298.9 (370.4)
SGA status^a^	24 (22)		12 (25)	12 (20)
Multiple birth	24 (22)		11 (22)	13 (21)
5 min Apgar, *M* (SD) (*n* = 109)	6.4 (1.8)	1–9	6.4 (1.6)	6.5 (2.0)
Arterial pH, *M* (SD) (*n* = 94)	7.3 (0.1)	7.0–7.4	7.3 (0.1)	7.3 (0.1)
Age of the mother, years, *M* (SD)	33.1 (5.2)	20–48	33.0 (5.4)	33.1 (5.1)
Chorioamnionitis	12 (11)		4 (8)	8 (13)
Toxemia	32 (29)		16 (33)	16 (26)
Section	68 (62)		34 (69)	34 (56)
Postnatal corticosteroids	16 (15)		7 (14)	9 (15)
Respiratory distress syndrome	35 (32)		20 (41)	15 (25)
Bronchopulmonary dysplasia at 28 days (*n* = 95)	43 (45)		21 (48)	22 (43)
Bronchopulmonary dysplasia at 36 gestational weeks (*n* = 97)	18 (19)		10 (22)	8 (15)
Sepsis	14 (13)		6 (12)	8 (13)
Necrotizing enterocolitis	3 (3)		1 (2)	2 (3)
Patent ductus arteriosus	9 (8)		4 (8)	5 (8)
Intraventricular hemorrhage	22 (20)		11 (22)	11 (18)
Grade I	15 (14)		7 (14)	8 (13)
Grade II	6 (6)		3 (6)	3 (5)
Grade III	1 (1)		1 (2)	0 (0)
Retinopathy of prematurity (*n* = 50)	13 (26)		7 (27)	6 (25)
Treated retinopathy of prematurity (*n* = 50)	4 (8)		1 (4)	3 (13)

^a^Small for gestational age (birth weight > 2 SD below the mean according to the age- and sex-specific Finnish growth charts)

### Measures

Development of VP children was assessed at 2 years of CA (mean in months, 24.2; SD, 0.2; min.–max., 23.4–24.8). Cognitive skills were assessed using the nonverbal cognitive scale of PARCA-R [40, 41], which includes 34 items regarding, e.g., spatial abilities, symbolic play, and memory (max. score 34). Weak skills were defined by < 10th percentile according to the original sex-specific norms [40]. The original standardization sample (*N* = 6402) from the UK consisted of 94.3% full-term, 5.4% late to moderately preterm, and 0.3% VP children [42]. The validity of PARCA-R has been verified [43–46].

The original PARCA-R also includes sections to measure language development. The vocabulary checklist included in PARCA-R is originally the UK short-form version of the MacArthur Communicative Development Inventories (CDI-SF [40]). We used the Finnish version, FinCDI-SF Toddler version [47] (adapted from the original American short-form versions of the CDI [39]) which is targeted for children aged 18–24 months. It contains a 100-word checklist, with one score per each word in the child’s expressive vocabulary. FinCDI-SF has been normed in full-term Finnish-speaking children (weak skills, < 10th percentile; score, < 12), and its validity has been confirmed [48].

Emerging syntactic skills were assessed using 12 questions of the PARCA-R sentence complexity subscale [40]. This includes 12 sentence pairs from which parents choose the one that best reflects their child’s linguistic skills (max. score 24). As there are no norms or clinical cutoffs, we defined weak skills by score 0 (minimal sentence complexity or no combined words). Among 2-year-old full-term Finnish children, approximately 90% combine words [35]. Thus, the weakest 10th percentile consists of children not yet using word combinations.

The CSBS-DP-ITC contains 24 multiple choice questions (max. score 57) divided into the following three subscales: communication (13 questions, max. score 26); expressive speech (5 questions, max. score 14); and symbolic (6 questions, max. score 17). The Finnish version has been translated and slightly adapted from the English version [38] and normed and validated for Finnish children [49] (weak skills, < 10th percentile; total score, < 49; communication, < 21; expressive speech, < 12; symbolic, < 15).

In this study, reliability analysis for the instruments indicated acceptable or excellent internal consistency (PARCA-R cognitive scale, Cronbach’s *α* = 0.79; FinCDI-SF, *α* = 0.99; PARCA-R sentence complexity subscale, *α* = 0.92; CSBS-DP-ITC, *α* = 0.83). Associations between the methods are presented in Supplementary Table 1.

### Statistical analysis

Data were analyzed using IBM SPSS Statistics 28.0. The differences between raw mean scores of VP children and norming samples were tested using Welch’s *t*-test. Boys and girls were tested separately in the PARCA-R cognitive scale. Distributions in continuous variables appeared non-normal through visual inspection, and therefore group differences within our sample were tested using nonparametric methods. In detail, sex differences in background characteristics were tested with Mann–Whitney *U* test (continuous variables) and *χ*^2^ test (categorical variables). The raw scores of boys and girls in cognitive and language scales were compared with Mann–Whitney *U* test. In addition, according to separate (PARCA-R) or combined (FinCDI-SF, CSBS-DP-ITC) norms for boys and girls, children were divided into percentile groups, and their distributions across groups were compared with *χ*^2^ test. False discovery rate (FDR) correction was applied using the Benjamin–Hochberg method to adjust for multiple testing (targeted FDR level, 0.05).

Explorative factor analysis (generalized least squares (GLS) method with direct oblimin rotation) was used to assess which variables are compatible when forming the profile groups based on typical/weak cognition/language. Factor analysis was conducted for the following six variables: the cognitive and sentence complexity scales of PARCA-R, FinCDI-SF, and the three subscales from CSBS-DP-ITC. To further examine the formed profile groups, we assessed how sex, perinatal risk factors, and medical complications predicted membership in a profile group using multinomial logistic regression. The composite variables of perinatal risk (gestational weeks < 28, SGA status, 5-min Apgar ≤ 3, or combinations thereof) and medical complications (respiratory distress syndrome, bronchopulmonary dysplasia at 36 gestational weeks, sepsis, necrotizing enterocolitis, intraventricular hemorrhage (Grade > 1), treated retinopathy of prematurity, or combinations thereof) were formed to identify those with known developmental risk. Profile groups served as the dependent variable and each background characteristic or composite variable as the independent variable in their respective regression models. FDR correction was applied.

## Results

### Level of cognitive and language skills

The prevalence of weak skills varied across the cognitive and language domains between 16 and 34% (Table 2). Based on PARCA-R sentence complexity score, 26% of the children used one-word utterances only. VP children in our sample had weaker skills in PARCA-R cognitive scale (*p* < 0.001), FinCDI-SF (*p* = 0.003), and CSBS-DP-ITC (*p* < 0.001) than the norm samples of the measures. The proportion of children in norm-based percentile groups are presented in Table 3. Depending on the measure, 43–72% of VP children scored < 25th percentile.

**Table 2:** Descriptive statistics for raw scores in cognitive and language scales

Measure	Mean (SD)	Median	Min.–max	Weak skills (< 10th percentile) *n* (%)	Mean (SD) for boys	Mean (SD) for girls
**Cognitive skills**						
PARCA-R^a^ cognitive scale	24.4 (4.2)	24.5	6–33	CA^b^: 34 (31) CH^c^: 46 (42)	24.6 (4.0)	24.3 (4.4)
**Language skills**						
FinCDI-SF^d^	43.6 (29.3)	40.5	0–100	21 (19)	37.2 (29.9)	48.7 (28.1)
PARCA-R sentence complexity	9.9 (7.6)	12	0–24	28 (26)^e^	8.6 (7.8)	10.9 (7.3)
CSBS-DP-ITC^f^ total score	49.3 (5.9)	51	10–57	37 (34)	48.8 (5.0)	49.7 (6.6)
Communication	21.8 (3.0)	22	3–26	22 (20)	22.0 (2.6)	21.6 (3.3)
Expressive speech	11.9 (2.8)	13	1–14	34 (31)	11.4 (3.0)	12.3 (2.5)
Symbolic	15.6 (1.6)	16	6–17	17 (16)	15.4 (1.5)	15.8 (1.7)

^a^Parent Report of Children’s Abilities–Revised

^b^Corrected age (age calculated from the expected date of delivery)

^c^Chronological age (age calculated from the date of birth), age- and sex-specific norms were used (age blocks, 25.5–26.5 months [*n* = 68] and 26.5–27.5 months [*n* = 42])

^d^Finnish short-form version of the MacArthur-Bates Communicative Development Inventories

^e^Weak skills in the sentence complexity subscale of PARCA-R were defined by score 0

^f^Communication and Symbolic Behavior Scales Developmental Profile–Infant–Toddler Checklist

**Table 3:** Number and proportion of children (*N* = 110) in each percentile group in PARCA-R cognitive scale, FinCDI-SF, and CSBS-DP-ITC total score

Measure	< 10th percentile	10–24th percentile	25–75th percentile	76–90th percentile	> 90th percentile
**Cognitive skills**					
PARCA-R^a^ cognitive scale	34 (31)	32 (29)	36 (33)	7 (6)	1 (1)
**Language skills**					
FinCDI-SF^b^	21 (19)	26 (24)	50 (46)	5 (5)	8 (7)
CSBS-DP-ITC^c^ total score	37 (34)	42 (38)	31 (28)^d^		

All values are *n* (%)

^a^Parent Report of Children’s Abilities–Revised

^b^Finnish short-form version of the MacArthur-Bates Communicative Development Inventories

^c^Communication and Symbolic Behavior Scales Developmental Profile–Infant–Toddler Checklist

^d^25–100th percentile (in CSBS-DP-ITC, percentile groups > 25% were not separable due to the ceiling effect in the norming sample)

### Sex differences in cognitive and language skills

There were no sex differences in the raw scores of PARCA-R cognitive scale (*p* = 0.50), PARCA-R sentence complexity subscale (*p* = 0.06), or CSBS-DP-ITC total score (*p* = 0.14) or its subscales (communication, *p* = 0.62; expressive speech, *p* = 0.09; symbolic, *p* = 0.11) (Table 2). Although girls scored higher than boys in FinCDI-SF (*p* = 0.03), the *p*-value was not significant after FDR correction.

**Fig. 1:**
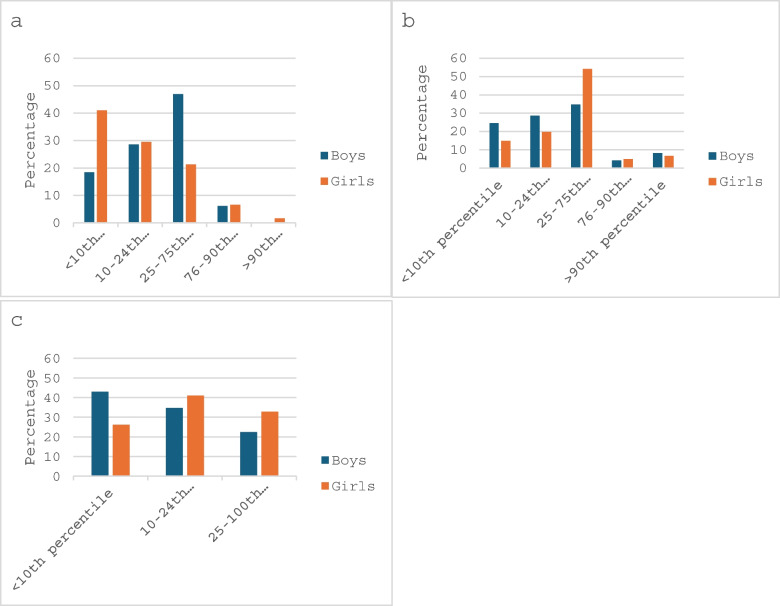
Proportion (%) of boys and girls in norm-based percentile groups in PARCA-R (sex-specific norms (**a**)), FinCDI-SF (joint norms for both sexes (**b**)), and CSBS-DP-ITC (joint norms for both sexes (**c**)). In CSBS-DP-ITC, percentile groups > 25% were not separable due to the ceiling effect in the norming sample

Distributions of boys and girls in norm-based percentile groups are shown in Fig. 1. A sex difference between distributions was found for PARCA-R cognitive scale (*p* = 0.03, Panel 1a) but not for FinCDI-SF (*p* = 0.33, Panel 1b) or CSBS-DP-ITC (*p* = 0.17, Panel 1c). However, the difference was not significant after FDR correction.

### Profile groups

**Fig. 2:**
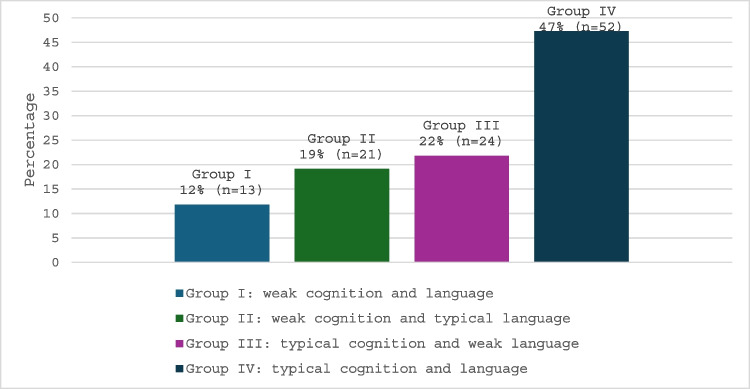
Proportion of children (*N* = 110) in profile groups based on cognitive and language skills (weak: < 10th percentile, typical: ≥ 10th percentile)

In the explorative factor analysis, a two-factor model offered the best fit. Factor 1 (cognition) consisted of PARCA-R cognitive scale and communication and symbolic subscales of CSBS-DP-ITC, and factor 2 (language) of FinCDI-SF, sentence complexity subscale of PARCA-R, and expressive speech subscale of CSBS-DP-ITC. To formulate a distinctive measure for cognition in factor 1, we chose to include only the PARCA-R cognitive scale and exclude subscales that also assessed linguistic skills. Weak cognitive skills were defined by < 10th percentile. In turn, language skills were defined by factor 2 with all its three scales that exclusively assessed linguistic skills, and weak skills by weak skills in any of these (< 10th percentile in FinCDI-SF or the expressive speech subscale of CSBS-DP-ITC, or score 0 in the sentence complexity subscale of PARCA-R). Four profile groups were formed (Group I: weak cognition and language; Group II: weak cognition and typical language; Group III: typical cognition and weak language; Group IV: typical cognition and language). Approximately half of the children were developing typically; 12% had weak skills in both domains and 41% had weak skills in only cognition or language (Fig. 2).

### Differences in background characteristics between profile groups

Background characteristics of the profile groups are shown in Table 4. Compared with Group IV, Group II had a larger proportion of girls (*p* = 0.04), and children in Groups II (*p* = 0.04) and III (*p* = 0.01) were more likely to have at least one perinatal risk factor. Although having at least one medical complication was not associated with profile group membership, children in Group III were more likely to have more than one medical complication (*p* = 0.04). The prevalence of specific background characteristics in Groups I–IV is presented in Supplementary Table 2.

**Table 4:** Background characteristics of the VP children in four profile groups

Characteristic	*n* (%) for Group I^a^ (*n* = 13)	*n* (%) for Group II^b^ (*n* = 21)	*n* (%) for Group III^c^ (*n* = 24)	*n* (%) for Group IV^d^ (*n* = 52)
Male sex	5 (39)	4 (19)*	16 (67)	24 (46)
Perinatal risk^e^	5 (39)	11 (52)*	14 (58)*	14 (27)
Medical complication^f^	4 (31)	8 (38)	14 (58)	21 (40)
> 1 medical complication^g^	2 (15)	3 (14)	7 (29)*	5 (10)

^a^Weak cognition and language

^b^Weak cognition and typical language

^c^Typical cognition and weak language

^d^Typical cognition and language

^e^Gestational weeks < 28, 5 min Apgar ≤ 3, small for gestational age status (SGA; birth weight > 2 SD below the mean according to the age- and sex-specific Finnish growth charts), or combinations thereof; composite variable: 1 = at least one perinatal risk factor, 0 = no perinatal risk or missing data

^f^Respiratory distress syndrome, bronchopulmonary dysplasia at 36 gestational weeks, sepsis, necrotizing enterocolitis, intraventricular hemorrhage (Grade > 1), treated retinopathy of prematurity, or combinations thereof; composite variable: 1 = at least one identified medical complication, 0 = no medical complication or missing data

^g^Respiratory distress syndrome, bronchopulmonary dysplasia at 36 gestational weeks, sepsis, necrotizing enterocolitis, intraventricular hemorrhage (Grade > 1), treated retinopathy of prematurity, or combinations thereof; composite variable: 1 = more than one identified medical complication, 0 = one or no medical complication or missing data

*p < 0.05. Associations between background characteristics and profile group membership were examined using separate multinomial logistic regression models (one predictor per model), with the profile group as the dependent variable and Group IV as the reference

## Discussion

We investigated the applicability of brief parental report instruments in cognitive and language skill assessment of VP children at 2 years of CA, and the possible sex differences in cognition and language skills of VP children. Generally, VP children had weaker cognitive and language skills than the norming samples of the measures, which consisted mainly of full-term children. No sex differences in cognitive or language skills were detected after statistical corrections. Approximately half of the VP children had typical cognition and language and approximately 10% had weak skills in both domains. A considerable proportion of children had weak skills in only cognition or language. Compared with the typically developing group, children with at least one perinatal risk factor were more likely to have weak skills in either one of the domains. In addition, girls were more likely to belong to the group of weak cognition and typical language, while children with typical cognition and weak language more likely had more than one medical complication.

In our study, 31% of VP children had weak (< 10th percentile, comparable to < − 1.28 SD in normal distribution) cognitive skills (expected proportion for cognitive delay, < − 1 SD, in the general population 16% [22]). In another study using PARCA-R [22], 34% of VP children had cognitive scores < − 1 SD. These comparable values suggest that PARCA-R provides valid information on the cognitive development of VP children at this age. Comparable values have also been derived from studies using formal tests to assess the cognitive skills of VP children at age 2 years. For example, 35% of VP children tested with Bayley-III scored < − 1 SD based on local term-born reference data [50]. Although the results of different studies are not fully comparable (e.g., due to different cutoffs), the results indicate that PARCA-R can detect cognitive delay in VP children.

In lexical skills, 19% of VP children had a weak score in FinCDI-SF. Parallel results were found in two studies with Finnish-speaking VP children when using the long-form version, CDI-LF (18% [21] and 15% [29]). In one study [51] with CDI-SF, the prevalence of weak skills was clearly higher (39.7%), and the highest risk was among those with high perinatal risk and mothers with low education, suggesting that background characteristics may influence the lexical skills of VP children. Overall, weak lexical skills appear to be more common in VP than full-term children and are detectable with brief validated parental report instruments. Consistently, a recent meta-analysis [52] showed weaker language skills in preterm compared with full-term children measured with CDI-LF up to 18 months; our study complements these results by assessing 2-year-old VP children. Furthermore, based on PARCA-R sentence complexity score, 26% of VP children used only one-word utterances. Nearly parallel results were found in three studies where 21% [53], 24% [54], and 24.6% [51] of VP children were not combining words at this age. These results suggest that not yet combining words at 2 years of CA is approximately at least twice as common in VP than in full-term children [35].

In our study, the mean value of CSBS-DP-ITC total score was 49.3, which is close to the 10th percentile value 49 of the Finnish norms [49]. In CSBS-DP-ITC, a ceiling effect was found in Finnish full-term children aged 2 years [49]; accordingly, the method has been proposed to be better suited for children aged < 2 years. Our findings differ from this view and suggest that CSBS-DP-ITC is indeed suitable for use in children aged 2 years with increased risk for language delays.

In the present study, no sex difference was found in raw scores of the PARCA-R cognitive scale, which is consistent with the results of Romeo et al. [18]. Sex difference in cognition has been reported in full-term children at early ages at least in some studies [14–16]. In addition, some previous studies reported a sex difference in cognitive skills in VP children at 2 years of CA as well [55, 56]. One possible explanation for our finding of missing sex difference in cognition could be lower survival of boys at very early gestational weeks [57, 58]. This may lead to a more selected group of boys, where only the more resilient and possibly developmentally faster boys reach age 2 years. In our study, VP girls did not outperform boys unlike the mainly (94.3%) full-term children in the PARCA-R norming sample, which questions the suitability of sex-specific norms in preterm populations. Based on our results, sex-specific norms should be used with caution with VP children as they may not be applicable in this population.

Although the girls in our sample generally had a larger expressive vocabulary than the boys, this difference disappeared after statistical correction. Sex difference in language skills favoring girls is a typical finding in full-term children at this age [17]. Consistently, in some studies with 2-year-old VP children, girls outperformed boys in language skills [8, 59], specifically in expressive vocabulary [60, 61], although not all studies have confirmed this [11, 12]. Generally, male sex is considered a risk factor for language delays, but our findings indicate that the risk may affect both sexes among VP children.

The identification of profile groups based on typical/weak cognition/language offers methodologically novel and clinically important information. Only approximately half of the participants scored typically in both cognition and language, which emphasizes the need for clinical follow up in this group. Most importantly, 41% of the children had weak skills in only one of the domains. This indicates that although cognition and language are often linked together, weak skills can be found solely in either domain. Thus, there is a clear need to measure both developmental domains separately to detect delayed development.

A novel finding of the present study is that background characteristics predicted membership in certain profile groups based on typical/weak cognition/language. Compared with the typically developed group, perinatal risk factors were associated with both weak cognition and typical language and vice versa. VP girls were more likely to have weak cognition and typical language compared with boys which may reflect the sex-specific norms of PARCA-R. In addition, children with more than one medical complication were more likely to have typical cognition and weak language. The effect of perinatal risk factors and medical complications on the development of VP children has been reported previously (e.g., SGA status [6, 62], lower gestational weeks [3, 63], intraventricular hemorrhage [8, 64]) but not in the context of profile groups as in this study. Further research is needed to verify these findings and clarify the mechanisms behind this effect.

### Strengths and limitations

This study provided novel information on the cognitive and language skills of VP children and applicability of brief parental report instruments in their assessment at 2 years of CA. Cognitive and language skills were assessed separately but in parallel, which revealed clinically important phenomena about the development of these domains in VP children. In addition, the comparison of sex differences, different analyses of the results, and information about background characteristics offered diverse information. The sample size was fairly large.

Despite the large sample, an even larger sample would have allowed for more detailed statistical analyses and higher statistical power to also detect differences in the smallest profile group. In addition, 28% of eligible children were not included in the final sample as the parents could not be contacted, declined participation, or did not complete the brief parental report instruments. Participants and non-participants could not be compared in background characteristics and therefore potential selection bias could not be evaluated. Although the validity and reliability of the methods used in the present study have been verified, parallel use of formal tests would have allowed for even more detailed validation and comparison. While it is a strength that the methods were normed, differing norming samples complicated the interpretation of the results. When forming the profile groups, the language measure included only expressive language. Information on receptive language development would have provided even more comprehensive information on language ability [65, 66]. In addition, testing the effect of maternal education level on cognitive and language development of VP children would have added depth to our findings. However, the norming studies of the FinCDI-SF and CSBS-DP-ITC did not reveal a significant effect of maternal education level on language development of full-term Finnish children at age 24 months. Lastly, although associations between profile groups and background characteristics were examined, direct causal conclusions cannot be drawn.

### Clinical implications

These findings suggest that the brief parental report instruments PARCA-R, CDI-SF, and CSBS-DP-ITC can be used as screening tools in clinical settings to detect weak skills in cognition and language in VP populations at 2 years of CA. The findings emphasize the importance of assessing cognition and language separately to identify individual needs of support. Sex-specific norms when assessing early cognition and language of VP children at this age should be used with caution.

## Conclusions

This study provided novel information on the utility of validated screening tools on cognitive and language assessment and the possible lack of sex difference in these domains in VP children aged 2 years. The results suggest that brief parental report instruments are useful in detecting delayed cognitive and language development among VP children in clinical work. VP children had significantly weaker cognitive and language skills than full-term peers in the norming samples, which shows the importance of assessing and supporting the development of VP children at an early age. No sex difference was detected in cognitive or language development in the present sample of VP children. Our results emphasize that weak skills can be detected in only cognitive or language skills or in both, highlighting the importance of separate cognitive and language assessment.

## Supplementary Information

Below is the link to the electronic supplementary material.

ESM 1 Supplementary Material 1 (DOCX 35.4 KB)

## Data Availability

The data cannot be shared openly to protect the privacy of the study participants.

## Abbreviations

CA: Corrected age
CSBS-DP-ITC: Infant-Toddler Checklist of Symbolic Behavior Scales Developmental Profile–Infant Toddler Checklist
FinCDI-SF: Finnish short-form version of the MacArthur Communicative Development Inventories
PARCA-R: Parent Report of Children’s Abilities–Revised
SD: Standard deviation
SGA: Small for gestational age
VP: Very preterm
