# Supplementary material for: Cognition and language of 2-year-old very preterm children measured using brief validated parental report instruments: a cross-sectional study
Source: Eur J Pediatr. 2026 Jun 23;185(7):520. doi: 10.1007/s00431-026-07163-3 (PMC13290927; doi:10.1007/s00431-026-07163-3)
Supplement: Supplementary file 1 — Supplementary Material 1 (DOCX 35.4 KB) [file 431_2026_7163_MOESM1_ESM.docx]

**Supplementary Fig. 1** Recruitment process of the children and reasons for exclusion

**STEP-WISE REASONS FOR EXCLUSION BY 2 YEARS OF CORRECTED AGE**

Children born in April 2020-June 2022 at gestational age <32 weeks, birth weight ≤1500 g, or both and treated at the Neonatal Intensive Care Unit of Helsinki University Hospital

**n = 319**

Exitus

**n = 25**

Children living in families with language other than Finnish

**n = 119**

Children living outside the hospital district of Helsinki University Hospital*

**n = 18**

Children having major neurological impairment

**n = 5**

Children that met the inclusion criteria

**n = 152**

Families could not be contacted

**n = 23**

Families declined participation

**n = 6**

Parents did not complete the parental reports

**n = 13**

Children included in the study

**n = 110**

*Altogether 20 children lived outside the hospital district of Helsinki University Hospital. Two of them were recruited and included as the family had moved, which did not occur during the recruitment process.

**Supplementary Table 1** Associations between the methods used in the present study

| **Variables** | **PARCA-R^a^:**  **Cognition** | **FinCDI-SF^2^** | **PARCA-R:**  **sentence complexity** |
| --- | --- | --- | --- |
| **FinCDI-SF^b^** | 0.42** |  |  |
| **PARCA-R: sentence complexity** | 0.33** | 0.84** |  |
| **CSBS-DP-ITC^c^: total score** | 0.60** | 0.59** | 0.53** |
| **CSBS-DP-ITC: communication** | 0.54** | 0.29* | 0.23 |
| **CSBS-DP-ITC: expressive speech** | 0.33** | 0.68** | 0.66** |
| **CSBS-DP-ITC: symbolic** | 0.62** | 0.48** | 0.38** |

Pearson’s correlation co-efficient values (r-values) are presented. In CSBS-DP-ITC, correlations are presented for total score and three subscales.

^a^PARCA-R = Parent Report of Children’s Abilities – Revised
^b^FinCDI-SF = Finnish short-form version of the MacArthur-Bates Communicative Development Inventories
^c^CSBS-DP-ITC = Communication and Symbolic Behavior Scales Developmental Profile – Infant-Toddler Checklist
**p*<0.05
***p*<0.001

**Supplementary Table 2** Background characteristics of the very preterm participants in four profile groups

| **Characteristic** | **n (%) for Group I^a^ (n=13)** | **n (%) for Group II^b^ (n=21)** | **n (%) for Group III^c^ (n=24)** | **n (%) for Group IV^d^ (n=52)** |
| --- | --- | --- | --- | --- |
| Gestational age (weeks), M (SD) | 30.5 (1.7) | 29.8 (2.6) | 29.3 (2.9) | 30.0 (2.0) |
| Birth weight (grams), M (SD) | 1363.5 (354.0) | 1231.2 (417.9) | 1188.6 (377.9) | 1358.9 (316.4) |
| SGA status^e^ | 3 (23) | **9 (43)*** | 6 (25) | 6 (12) |
| Multiple birth | 0 (0) | 5 (24) | 3 (13) | 16 (31) |
| 5 min Apgar, M (SD) | 6.7 (1.6) | 6.9 (1.7) | 6.1 (2.1) | 6.3 (1.7) |
| Arterial pH, M (SD) | 7.3 (0.1) | 7.3 (0.1) | 7.3 (0.1) | 7.3 (0.1) |
| Age of the mother (years), M (SD) | 33.6 (5.2) | 33.4 (4.0) | 33.9 (6.2) | 32.4 (5.2) |
| Chorioamnionitis | 1 (8) | 2 (10) | 3 (13) | 6 (12) |
| Toxemia | 4 (31) | 6 (29) | **11 (46)*** | 11 (21) |
| Section | 4 (31) | 9 (43) | 7 (29) | 22 (42) |
| Postnatal corticosteroids | 1 (8) | 4 (19) | **7 (29)*** | 4 (8) |
| Respiratory distress syndrome | 2 (15) | 6 (29) | 11 (46) | 16 (31) |
| Bronchopulmonary dysplasia at 28 days | 4 (31) | 9 (43) | 11 (46) | 19 (37) |
| Bronchopulmonary dysplasia at 36 gestational weeks | 1 (8) | 4 (19) | 6 (25) | 7 (14) |
| Sepsis | 1 (8) | 3 (14) | 4 (17) | 6 (12) |
| Necrotizing enterocolitis | 1 (8) | 0 (0) | 2 (8) | 0 (0) |
| Patent ductus arteriosus | 1 (8) | 3 (14) | 2 (8) | 3 (6) |
| Intraventricular hemorrhage | 3 (23) | 4 (19) | 5 (21) | 10 (19) |
| Grade I | 1 (8) | 3 (14) | 4 (17) | 7 (14) |
| Grade II | 2 (15) | 1 (5) | 1 (4) | 2 (4) |
| Grade III | 0 (0) | 0 (0) | 0 (0) | 1 (2) |
| Retinopathy of prematurity | 1 (8) | 4 (19)* | 5 (21) | 3 (6) |
| Treated retinopathy of prematurity | 0 (0) | 1 (5) | 2 (8) | 1 (2) |

^a^Group I = weak cognition and language
^b^Group II = weak cognition and typical language
^c^Group III = typical cognition and weak language
^d^Group IV = typical cognition and language
^e^SGA status = small for gestational age (birth weight more than 2 SD below the mean according to the age- and sex-specific Finnish growth charts)
**p*<0.05. Associations between background characteristics and profile group membership were examined using separate multinomial logistic regression models (one predictor per model), with the profile group as the dependent variable and Group IV as the reference.
